# Spontaneous Intracranial Vertebral Artery Dissection: A Rare Cause of Ischemic Stroke

**DOI:** 10.3390/jcdd12050187

**Published:** 2025-05-15

**Authors:** Marialuisa Zedde, Rosario Pascarella

**Affiliations:** 1 Neurology Unit, Stroke Unit, Azienda Unità Sanitaria Locale-IRCCS di Reggio Emilia, Viale Risorgimento 80, 42123 Reggio Emilia, Italy; 2 Neuroradiology Unit, Ospedale Santa Maria della Misericordia, AULSS 5 Polesana, 45100 Rovigo, Italy; rosario.pascarella@aulss5.veneto.it

**Keywords:** vertebral artery, intracranial, intradural, dissection, DSA, MRI, MRA, CTA, vessel wall, stroke, mural hematoma, intimal flap

## Abstract

The dissection of the V4 vertebral artery (VA) is the most prevalent form of intracranial dissection, which can manifest either as ischemia or as a subarachnoid hemorrhage (SAH). Patient outcomes are significantly affected by their initial presentation; ischemic symptoms often indicate that the dissection remains primarily subintimal or within the medial layer, though it can occasionally extend to the basilar artery. In contrast, patients with ruptured VA dissection (VAD) experience a considerably higher mortality rate, as the dissection can reach the adventitial layer, heightening the risk of recurrent hemorrhage. It can show fluctuating imaging findings, making an accurate diagnosis and timely treatment essential. Currently, there are no established diagnostic criteria for VAD, and its diagnosis largely depends on imaging. The presence of intramural hematoma, identified via three-dimensional, black-blood, T1-weighted imaging, has been recognized as the most reliable indicator for diagnosing VAD and is crucial for establishing a definitive diagnosis. DSA remains a fundamental diagnostic technique not only in hemorrhagic patients but also in ischemic patients. The medical treatment of ischemic patients has not yet been well defined, and evidence-based data are lacking. This review aims to summarize the main clinical, pathophysiological, and neuroradiological features of intracranial VAD presenting with ischemic stroke, providing to clinicians the available information in order to individualize the treatment.

## 1. Introduction

Cervicocranial artery dissection, characterized by a hematoma forming within the wall of either a cervical or intracranial artery, is a significant cause of stroke, particularly in children and young to middle-aged adults [1,2,3]. While extensive research has focused on extracranial cervical artery dissection [4,5,6,7,8,9,10,11,12], our understanding of isolated intracranial artery dissection (i.e., without cervical artery involvement) remains limited [4]. Early studies on intracranial artery dissection predominantly relied on autopsy reports, which often highlighted the most severe cases [13,14]. Several factors contribute to the scarcity of information regarding intracranial artery dissection. One such factor is the lower prevalence of these dissections compared to cervical artery dissections in non-Asian countries, where the largest patient cohorts for cervical artery dissection have been documented [9,11,12]. Additionally, patients with cervical artery dissection typically present with headache, neck pain, and ischemic stroke, and are primarily managed by neurologists. In contrast, intracranial artery dissection may also result in subarachnoid hemorrhage (SAH), which requires care from neurologists, as well as neurosurgeons and interventional neuroradiologists, each potentially possessing only a partial understanding of the condition. Consequently, there is currently no consensus on the diagnostic criteria or optimal treatment for intracranial artery dissection. In adults, dissections affecting the posterior circulation are the most common, with the dissection of the V4 segment of the vertebral artery (VA) being the most prevalent. Intracranial VAD can manifest in two primary forms: as focal neurological deficits resulting from vertebrobasilar artery ischemia or as SAH. The initial presentation significantly influences patient outcomes. When symptoms are ischemic, the dissection tends to remain primarily subintimal or confined to the medial layer, although it may occasionally extend to the basilar artery. In contrast, patients with ruptured VA dissection (VAD) experience a markedly higher mortality rate, as the dissection plane reaches the adventitial layer, thus increasing the risk of recurrent hemorrhage [15]. This review aims to summarize the main clinical and neuroradiological features of spontaneous intracranial VAD that presents symptomatically as ischemic stroke.

## 2. Epidemiology and Pathophysiology

While extensive investigations have focused on the dissection of extracranial cervical arteries, isolated intracranial artery dissection remains less well understood. Initial studies primarily concentrated on more severe cases, often derived from autopsy reports [13,14]. Intracranial artery dissections are less common than their cervical counterparts, particularly in non-Asian countries, where the most comprehensive cases of cervical dissection have been recorded. Patients experiencing cervical artery dissection typically present with symptoms such as headaches, neck pain, and ischemic strokes, whereas those with intracranial artery dissection may suffer from SAH and are frequently managed by a multidisciplinary team that includes neurologists, neurosurgeons, and interventional neuroradiologists. Nonetheless, no established consensus exists on diagnostic criteria or optimal treatment strategies [16].

The precise incidence of intracranial artery dissection is not well defined, but it is thought to be lower than that of cervical artery dissection, which range from 2.6 to 3.0 per 100,000 individuals annually in European populations [17,18]. A study conducted in France and Switzerland found that only 11% of dissections were located within the intracranial segment when patients were recruited through neurology departments [19]. In contrast, research from East Asia indicated that intracranial artery dissections represented 67–78% of cervicocephalic artery dissections, particularly within the vertebrobasilar territory [20,21,22]. Notably, intracranial artery dissections tend to affect the posterior circulation more frequently in adults, while in children, they are more often observed in the anterior circulation, likely due to a bias towards cases without SAH [23]. A study that combined data from neurosurgery and neurology departments reported that 54% of intracranial artery dissections led to SAH [24].

From a pathophysiological standpoint, the anatomical structure of intracranial arteries makes them more susceptible to dissection and subsequent SAH compared to cervical arteries. The intradural segments of the vertebral and internal carotid arteries have less robust supporting tissue, which can lead to subadventitial dissection and bleeding [25,26]. Specifically, dissections originating from intradural segments, such as the V4 segment of the vertebral artery, have a higher probability of causing SAH [27,28]. Intracranial arterial dissection is a rare cause of stroke, with the posterior circulation being more commonly affected than the anterior circulation in adults [29,30]. Among these, the intracranial VA is the most frequently involved artery [16]. Despite this prevalence, intracranial VAD is overall an uncommon cause of stroke and an even rarer cause of spontaneous SAH. The exact incidence of intracranial VAD has yet to be determined; however, vertebrobasilar dissection accounts for 3% to 7% of SAH cases [16].

Once considered quite rare, VAD has emerged as a significant contributor to neurological morbidity and mortality, with an estimated annual incidence of approximately 1–1.5 cases per 100,000 individuals [6,31,32,33,34,35,36,37,38,39]. The VA is involved in up to 81.6% of cases, making it the most commonly affected artery in spontaneous posterior circulation dissection [40]. The prognosis for patients with intracranial VAD remains uncertain, even with contemporary medical practices, largely due to the heightened risk of early rebleeding associated with this condition [36,41]. The mortality rate linked to intracranial VAD is notably high, ranging from 19% to 83% [42]. Importantly, VAD is a significant cause of stroke in younger populations, accounting for 10–25% of strokes in individuals aged 25 to 45 years [43]. VAD predominantly affects patients in their fourth decade of life, which is slightly older than the demographic typically impacted by carotid dissection, which is more prevalent in the second and third decades of life [6]. Additionally, advancing age appears to be a considerable risk factor for the development of VAD-associated ischemia, with individuals experiencing ischemic strokes averaging an age of 54.6 years [44,45]. In recent decades, treatment approaches have increasingly leaned towards neuro-interventional methods; however, debates continue regarding the best endovascular techniques to prevent rebleeding and the appropriate indications for revascularization surgery [46].

## 3. Anatomical Remarks

The VAs arise from the subclavian arteries and navigate through the foramina transversaria, starting at the C6 vertebra, before entering the foramen magnum, where they converge to form the basilar artery. Within the cranial cavity, prior to their unification with the basilar artery, the VAs give rise to significant cortical branches, particularly the posterior inferior cerebellar arteries (PICAs), as well as a varied number of perforating arteries that nourish the brainstem and upper spinal cord. The anatomy of the VA presents considerable variability, which can include duplications, fenestrations, accessory arteries, atypical origins and pathways, hypoplasia, aplasia, extradural origins of the PICA, the presence of an arcuate foramen, and enduring embryological connections between the anterior and posterior circulations [47]. Moreover, the anatomy of the perforating arteries is notably inconsistent. Some anatomical variations have been associated with VAD, although the connections with others remain ambiguous. Recognizing these anatomical differences is essential for developing tailored treatment plans for individual patients.

Typically, the VA emerges from the subclavian artery; however, one prevalent anatomical variant is its direct origin from the aortic arch, which is more frequently observed on the left side [48]. In certain cases, the VA may also originate from the common or external carotid arteries or from the thyrocervical trunk. A direct origin from the aortic arch has been linked to heightened hemodynamic stress on the vessel, increasing the likelihood of dissection. When the VA originates from the aortic arch, its diameter at the point of origin tends to be larger compared to when it stems from the subclavian artery [49]. As the VA traverses the dura, its size diminishes in relation to its extradural portion; nevertheless, its intracranial diameter remains comparable to that of a VA originating from the subclavian artery [49]. Despite these distinctions and similarities, the aortic origin of the vertebral artery has been correlated with a greater occurrence of V4 segment dissection [50]. Yamaura et al. [38] further classified the V4 segment into three subdivisions: V4 (1), located between C1 and the dural penetration; V4 (2), situated between the dural penetration and the PICA origin; and V4 (3), found between the PICA origin and the confluence with the contralateral VA. The authors noted that the VA was involved at V4 (3) in 8 out of 19 cases, and at V4 (2, 3) in 11 out of 19 cases, with none of the VADs showing the exclusive involvement of V4 (1) or V4 (2). Intracranial dissection can result in both ischemic events and SAH. Group 1 intracranial VADs, which are confined to the V4 segment without affecting the basilar artery, are the most prevalent. These often lead to dissecting aneurysms or pseudoaneurysms, resulting in SAH. Thrombotic events are also possible, as thrombi formed within dissecting aneurysms or pseudoaneurysms may embolize [51]. Less frequently, a VAD may extend to involve the basilar artery (Group 2), typically resulting in a subintimal hematoma, which leads to symptoms of brainstem ischemia, rather than SAH. Over time, these dissections can culminate in an ischemic stroke or SAH weeks or even months following the initial event [32].

Many studies reference the term “intracranial vertebral artery dissection”, yet only a handful provide a clear definition, often identifying it as a diameter difference of 0.3 mm or more between the two VAs [52]. The prevalence of intracranial VAD in the dominant versus non-dominant VA remains an open question. Dissection may arise due to abnormal hemodynamic effects on the vessel wall, including hypertension and supra- or infra-physiological wall shear stress (WSS) distributions. WSS, defined as the product of dynamic blood viscosity and the instantaneous change in blood velocity perpendicular to the vessel lumen, can be simplified in parabolic laminar flow to be proportional to blood velocity divided by vessel radius. A decrease in the vessel diameter may result in increased blood pressure and supraphysiological WSS, thereby heightening the risk of dissection on the non-dominant side. Additionally, variations in vessel diameter along a small, non-dominant VA can create significant WSS gradients that may initiate or worsen dissection [53]. Wall stretching serves as another hemodynamic stressor affecting blood vessels. According to Young–Laplace’s law, an increase in blood volume within larger diameter vessels can elevate wall tension, raising the risk of complications, particularly on the dominant side. However, the impact of increased vessel radius on wall tension may be offset by a decrease in blood pressure [53]. Sudden stretching could also act as a potential mechanism for arterial dissection [16,41], impacting both dominant and non-dominant sides.

In a radiological study by Dzierzanowski et al. [54], which examined the anatomy and variations of the V4 segment, it was found that the left VA was often larger than the right. Similar findings were reported in studies of VA anatomy in the cervical region [47]. However, clinical results have shown conflicting evidence, with VAD occurring bilaterally, as well as in both dominant [55] and non-dominant sides [56,57].

The tortuosity of the intracranial vertebrobasilar system is another potential risk factor for VAD. Hori et al. [55] defined tortuosity as a lateral displacement exceeding 2 mm of the basilar artery tip from a vertical plane aligned with the brainstem, which may shift toward either the dominant or non-dominant side. Comparisons between VAD patients and control groups indicated that VAD patients were more likely to exhibit tortuosity, particularly towards the contralateral side of the dominant vertebral artery. The presence of the posterior communicating artery (Pcom) has also been associated with VAD, although its anatomical influence on VAD development remains uncertain. Ariyada et al. [58] noted that patients with VAD and hemorrhage were more likely to have a hypoplastic Pcom, potentially increasing the workload on the posterior circulation. Conversely, Park et al. [57] found a correlation between Pcom presence and ruptured VAD, rather than unruptured cases. A literature review by Vogels et al. [59] underscored that the anatomy of VA perforators is a relatively uncharted domain, with current imaging methods proving insufficient to accurately visualize this anatomy. At varying distances from the vertebrobasilar junction, the VA gives rise to a small trunk that, in conjunction with a trunk from the contralateral VA, forms the anterior spinal artery (ASA). A cadaveric study [60] consistently demonstrated that the origin of the ASA was distal to the PICA in all the specimens analyzed. Documented variations include asymmetries in trunk size, unilateral trunks, and duplications of the ASA. Generally, posterior spinal arteries (PSAs) arise directly from the VAs in most cases (V3 or V4 segments) but may also emerge from the PICA [61,62]. Perforators from the PSA can ascend rostrally or laterally to anastomose with those from the ASA, providing blood supply to the medulla [63].

The anatomy of VA perforators shows significant variability, with origins from the VA itself, the trunks forming the ASA, or directly from the ASA [60]. These perforators may supply different regions of the medulla and can establish anastomoses with perforators from the basilar artery or anterior inferior cerebellar artery (AICA), though right-to-left anastomoses are uncommon [64]. In rare instances, the VA may lack perforators between its entry into the dura mater and the point where it gives rise to the PICA [65].

The PICA primarily originates from the intradural VA but can also arise from the extradural VA (15%), other cervical vessels, or a common trunk with the AICA [65]. The PICA may be singular or duplicated, providing a unilateral or bilateral supply to the hemispheric cerebellum. The number of perforators it generates varies. When originating from a common trunk with the AICA, the PICA does not supply perforators to the brainstem. When emerging extradural, the PICA ascends behind the medulla oblongata, typically issuing its perforating arteries posteriorly at the origin of the lateral spinal artery. Conversely, when it emerges intradurally from the VA, the PICA supplies perforators to the brainstem, which can be positioned anteriorly or laterally, depending on their point of origin [65].

## 4. Pathophysiology and Clinical Phenotypes

The exact pathophysiology of spontaneous VA dissection leading to ischemic stroke versus hemorrhage remains unclear. There is ongoing debate about whether the initial event in this process originates from a lesion in the intima or the media. In either case, the disruption of the internal elastic lamina occurs, allowing blood to accumulate in a pseudolumen beneath the adventitia. As the true lumen narrows due to the progressively thickening vessel wall, increased resistance causes blood to preferentially flow into the false lumen, worsening the situation. Eventually, the adventitia becomes distended to a critical point of rupture, leading to SAH. In a post-mortem analysis by Ro and Kageyama [66], which examined four histological lesions in dissected V4 segments—specifically adventitial ruptures, dilated lesions with disrupted internal elastic lamina, intimal tears with disrupted internal elastic lamina, and medial defects—it was found that medial defects were the most common along the length of the affected vessel. This finding led the authors to propose that medial degenerative disease and the subsequent disruption of the internal elastic lamina represent the initial event in the progression of intracranial VA dissection. A histological study of the normal VA in ten patients, conducted both before and after it enters the dura [67], indicated a progressive thinning of the media and adventitia, particularly following the origin of the PICA. In contrast, the thickness of the intima remained stable distally. Defects in the internal elastic lamina were observed in 11 vessels from six patients, particularly noted at the V3 segment.

Based on the classification system by Mizutani et al. [68], different pathomechanisms can lead to hemorrhagic, ischemic, or mass effect-related symptoms associated with arterial dissection. When blood enters the subintimal space due to a tear in the vessel wall, it may follow several distinct pathological courses (Table 1).

When high-pressure arterial blood enters the false lumen and dissects through the layers of the tunica media or between the media and adventitia, a dissecting aneurysm is formed [51]. An expanding hematoma can accumulate within the vessel wall as pro-thrombotic subendothelial areas are exposed to blood. This situation may lead to thrombotic phenomena, the occlusion of the parent vessel due to progressive stenosis or hematoma expansion, or SAH resulting from extension through the adventitia [42,66]. If the dissection progresses to involve all three layers of the vessel wall, a pseudoaneurysm may develop, resulting in the encapsulation of the extravascular hematoma [51]. Intracranial VAD can also present as hemorrhages due to the absence of an external elastic lamina and the reduced number of elastic fibers within the tunica media, with the pseudoaneurysm wall potentially rupturing into the surrounding subarachnoid space [67]. Pseudoaneurysms associated with VAD constitute approximately 28% of posterior circulation aneurysms and 3.3% of all intracranial aneurysms, with the potential to rupture either acutely or at a later time following dissection [69,70,71,72]. Furthermore, these lesions are the underlying cause in around 10% of all cases of non-traumatic SAH [66]. Pseudoaneurysms exhibit a high propensity for rupture, with studies showing that up to 73% present with SAH, while only 27% of symptomatic lesions demonstrate bulbar signs/symptoms or cerebellar ischemia [73]. A summary of VAD and its complications is illustrated in Table 2.

Collagen-related disorders associated with cerebrovascular arterial dissection include Ehlers–Danlos syndrome type IV [78], Loeys–Dietz syndrome, Marfan syndrome [79,80], and osteogenesis imperfecta type I [81]. Additionally, intracranial dissections have been noted in conjunction with conditions that impact the arterial media, such as fibromuscular dysplasia [82] and cystic medial necrosis. Genetic variants in the COL3A1, COL5A2, FBN1, and PDCD10 genes—responsible for encoding collagen types III and V, fibrillin-1, and a protein involved in apoptosis—have been linked to the development of intracranial dissection.

Other potential candidate genes and variants have also been identified, but further research is required to confirm these associations [83]. Additionally, pregnancy has been recognized as a distinct risk factor for dissection due to increased hemodynamic stress and hormonal fluctuations [84], with bilateral VAD being more common in pregnant patients [85].

The influence of vascular risk factors on intracranial dissection remains unclear. A study by Lee et al. in 2006 [86] found that hypertension, atherosclerosis, cholesterol disorders, and diabetes were less common in cases of intracranial dissection compared to cervical dissection. In contrast, another study [87] identified hypertension and age as contributing risk factors for intracranial VAD.

Intradural VAD is more likely to result in hemorrhage [88], as opposed to ischemic symptoms, which are typically associated with extradural dissection [89]. Patients experiencing a hemorrhage from an intracranial VAD often present with high-grade SAH and face an elevated risk of re-bleeding [90]. Prodromal symptoms, such as headaches or ischemic manifestations, are common and may be reported by up to 80% of patients [30], but they can be easily overlooked due to their nonspecific nature [16,66].

The clinical presentation of non-hemorrhagic intracranial VAD varies widely, ranging from ischemic stroke symptoms to nonspecific complaints like dizziness. The most frequently reported symptoms include headache, vertigo, and neck pain; however, no single symptom is specific enough to rule out or confirm the diagnosis. Notably, the absence of neck pain does not exclude VAD. Pain is the most common clinical symptom associated with VAD, largely due to the presence of pain nerve fibers (PNFs) in cerebral blood vessels, particularly the major cerebral arteries. These fibers originate from the trigeminal nerve—primarily the ophthalmic (V1) division—and cervical nerves C1, C2, and C3 [89].

Though uncommon, VAD can also cause spinal cord ischemia. While spinal cord infarction accounts for only 1% of all strokes, it occurs in 4–10% of VAD cases. Montalvo et al. documented a case of cervical spinal cord infarction at C3–C4 caused by right vertebral artery dissection, leading to anterior cord syndrome, quadriplegia, areflexia, urinary incontinence, and sensory loss up to the cervical level [91]. Another rare manifestation is spinal cord infarction with Brown–Séquard syndrome [92].

Symptoms of ischemia can manifest early or late, depending on the type of dissecting aneurysm. With Mizutani type 1 and 4 aneurysms, early rupture and a lack of collateral circulation will produce acute stroke-like symptoms and generally portend a poor clinical course. Transmural dissection may also present with severe, rapid ischemia secondary to occlusive intramural thrombosis. Partial thrombosis is observed surrounding fragmented areas of IEL, which can progress with recurrent dissection to produce late-onset symptomatic ischemia in the setting of Mizutani type 3 aneurysms [93].

In a large prospective study, up to 77% of patients diagnosed with VAD exhibited symptoms related to ischemia. Upon admission, 67% of these individuals had experienced an ischemic stroke, while an additional 10% presented after a TIA [19,44]. Approximately 88% of the patients with ischemic symptoms reported head and/or neck pain, and 5% experienced pulsatile tinnitus [19]. Notably, isolated occipital head and/or neck pain was documented in as many as 12% of the patients who suffered an ischemic event following a VAD [19]. There is considerable variability in the severity of neurological deficits among patients experiencing ischemic strokes due to VAD. The reports indicate a National Institutes of Health Stroke Scale (NIHSS) score ranging from 1 to 35, with an average admission score of 3. Among the patients who initially experienced a TIA (13%), the median time from the TIA to the stroke onset was 24 h, with a range from 1 h to 17 days [19]. Overall, more than 50% of the patients with intracranial VAD presented with brainstem ischemia. Wallenberg syndrome is frequently observed in these patients, reported in 26–43% of cases [94,95,96]. Additionally, an older age and the involvement of the basilar artery have been identified as independent predictors of poor outcomes in symptomatic intracranial VAD with ischemic presentation [21].

## 5. Neuroradiological Diagnosis

Intracranial VAD is often suspected in patients presenting with headache and/or neurological symptoms resulting from cerebral infarction. However, it frequently exhibits fluctuating imaging findings and symptoms, making accurate diagnosis and timely treatment crucial [55,97]. Currently, there are no established diagnostic criteria for vertebral artery dissection (VAD), and the diagnosis largely depends on imaging findings, which can present clinical challenges. For example, anatomical variations in the vertebral arteries can complicate the distinction between pathological stenosis and a non-dominant normal vertebral artery. The presence or absence of an intramural hematoma (IMH), as assessed by three-dimensional, black-blood, T1-weighted imaging, has been identified as the most reliable indicator for diagnosing VAD and is a crucial factor in confirming a definitive diagnosis. Identifying an IMH on MRI is particularly significant for evaluating the thrombus presence within the vessel walls during the acute phase of vascular dissection.

However, it is important to note that previous studies on vertebrobasilar dissecting aneurysms have reported IMH prevalence rates ranging from 32% to 100% [15,45,98,99], depending on the timing of the MRI. Historically, the presence of an aberrant arterial lumen was the standard criterion for diagnosing dissection in neuroimaging. Nonetheless, Ahn et al. [100] found that an IMH is more easily identifiable than the double lumen or intimal flap on MRI. The increased intensity of IMH is attributed to the electromagnetic effects of hemoglobin breakdown products. Similar to the double lumen and intimal flap associated with vascular dissection, an IMH is regarded as a definitive finding.

For an accurate diagnosis, susceptibility-weighted imaging and the use of contrast agents have also been investigated. Diagnostic criteria for intracranial dissection have been established in Asian populations and are summarized in Table 3 [101].

Debette et al. [16] proposed a summary of the common description of intracranial dissection on imaging and introduced a third category, i.e., possible intracranial dissection (Table 4 and Table 5).

**Figure 1:**
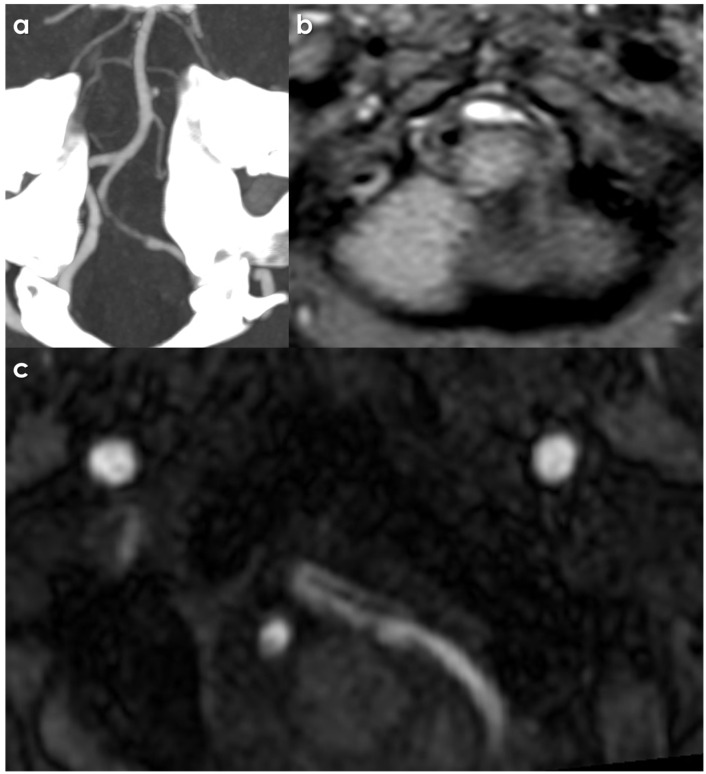
Intracranial left VAD. The CTA panel (**a**) shows a long-tapered stenosis, corresponding with the flow in the true lumen, as outlined by the presence of a mural hematoma and hyperintense T1 Dixon MRI panel (**b**) with an intimal flap (panel (**c**), MRA source image).

An example of CTA and MTI signs of intracranial VAD is illustrated in Figure 1.

DSA, particularly a comprehensive four-vessel study, remains a key diagnostic tool for cerebral arterial dissection. This imaging technique not only provides a detailed visualization of arterial structures but also allows for the assessment of blood flow dynamics. It is particularly valuable in cases of subarachnoid hemorrhage. Among the most characteristic findings of cerebral arterial dissection are the intimal flap and the double lumen. However, these features are not frequently observed in clinical practice. Studies suggest that CT angiography and MRI outperform conventional cerebral angiography in detecting intimal flaps and double-lumen formations [45,102,103,104]. Conversely, the pearl-and-string sign is a relatively specific indicator that is often identifiable through conventional cerebral angiography. Other common angiographic findings include fusiform aneurysmal dilations and stenotic segments at proximal and/or distal locations. The string sign, characterized by long-segment stenosis with a sawtooth appearance, is also a distinctive feature [15,89,105,106]. Retained contrast media within the pseudolumen may also be observed [107]. Over time, as the intramural hematoma resolves, these angiographic abnormalities tend to diminish. While fusiform aneurysmal dilation and tapered stenosis may sometimes be present, their occurrence is not always indicative of cerebral arterial dissection. Since the rapid injection of contrast media can exacerbate arterial dissection, the careful monitoring and control of the injection rate and the volume are essential to minimize potential complications. The main DSA findings are summarized in Table 6.

**Figure 2:**
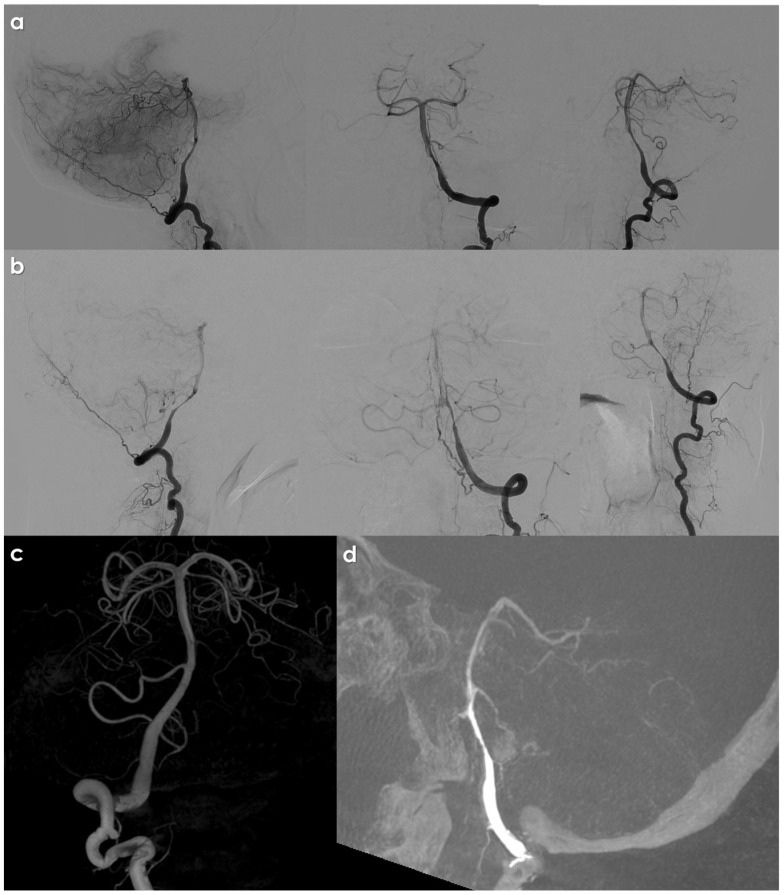
DSA from left (panels (**a**,**b**,**d**)); left (panel (**c**)) VA injection in a patient with left intracranial VAD, showing a long, irregular, tapered stenosis of the left V4 VA, immediately after PICA take off. Panel (**c**) shows the right VA reconstructed in volume rendering from a 3D rotational angiography acquisition. In panel (**d**), the source images of the 3D RA are reconstructed in an MIP/MPR protocol in a sagittal oblique plane passing though the left V4 VA.

Figure 2 illustrates an example of an intracranial VAD on DSA.

Multidetector CT angiography (MDCTA), utilizing 64–320 detector rows, is a fast, precise, and wide-field imaging modality that requires an intravenous contrast bolus injection (3.5–4.5 mL/s). Compared to catheter-based angiography, MDCTA offers a safer and more rapid alternative while still detecting many of the same key diagnostic features. Like conventional CT, modern multidetector CT enables 4D angiographic imaging, providing the dynamic visualization of vascular structures. It is effective in identifying arterial stenosis, occlusion, and fusiform dilations in patients with arterial dissection, comparable to catheter-based cerebral angiography. Additionally, MDCTA can facilitate the detection of double-lumen and intimal-flap signs, although these findings may not always be apparent [15,89,105,106,107,109]. A major advantage of CTA is its source imaging capability, which allows for the assessment of pseudolumen formation by visualizing the arterial wall thickness [110]. Moreover, studies suggest that CTA is superior to MRA for detecting arterial dissection [89,105,109,110,111]. The main issues related to CTA are summarized in Table 7.

MRI is a non-invasive and highly effective tool for diagnosing both acute infarction and cerebral arterial dissection. MRA provides the detailed visualization of arterial morphology, while three-dimensional volume imaging enhances the detection of intramural hematoma and double lumen formation, key indicators of arterial dissection. The main issues of MRI and MRA in intracranial dissection are summarized in Table 8.

MRI imaging is now the main non-invasive diagnostic technique for imaging intracranial dissection, as previously said. Some detailed clues about the use of MRI for imaging the vertebrobasilar arterial segments have been proposed.

Basi-parallel anatomic scanning MRI (BPAS-MRI) [117] is an MRI technique that can reveal the outer contour of the vertebrobasilar artery, even in the presence of occlusion. BPAS is a supplementary imaging modality that plays a supportive role in diagnosing cerebral arterial dissection; it utilizes a heavily T2-weighted imaging (T2WI)** technique that is both easy to perform and effective in providing useful diagnostic insights. This method generates coronal images of the vertebrobasilar artery, using thick slices (20 mm) to visualize the outer shape of the artery. BPAS is particularly effective in detecting fusiform aneurysmal dilations caused by arterial dissection, a feature that may not be identifiable using MRA alone. Although BPAS cannot provide a definitive diagnosis of arterial dissection by itself, it is a valuable screening tool for detecting potential dissection [118]. Additionally, BPAS helps in differentiating arterial stenosis or occlusion resulting from dissection from the hypoplasia of the vertebral arteries, which is a distinction that is challenging to make with MRA alone [118]. BPAS-MRI, when combined with time-of-flight (TOF) MRA, CTA, and/or digital subtraction angiography (DSA), enables the visualization of the inner vessel contours, aiding in the differentiation between congenital dysplasia, arteriosclerosis, and dissecting aneurysms within the vertebrobasilar system [118,119]. The 3.0 T conventional high-resolution MRI can effectively distinguish intracranial arterial walls and lumens from surrounding tissues with a resolution of less than 1 mm (ranging from 0.2 to 0.9 mm). This advanced imaging technique offers vital insights into the structure of the vascular wall and any occluded segments that may not be detected by other methods, such as time-of-flight magnetic resonance angiography (TOF-MRA), CTA, or DSA [120]. Conventional high-resolution MRI at 3.0 T is routinely used to diagnose conditions such as arterial dysplasia, atherosclerosis, arterial dissection, moyamoya disease, central vasculitis, and reversible vasoconstriction syndrome in the vertebrobasilar arteries [121] and has gained widespread acceptance for wall imaging. However, this method requires a 3.0 T MRI, entails prolonged scanning times, and may not be suitable for patients who cannot tolerate the procedure due to significant neurological deficits.

A recent study compared BPAS-MRI with conventional high-resolution MRI [117], evaluating the effectiveness of BPAS-MRI in diagnosing lesions in patients with abnormalities of the intracranial segment of the vertebrobasilar artery, as identified on TOF-MRA. The results revealed that for vertebrobasilar arteriosclerosis, the sensitivity, specificity, positive predictive value, negative predictive value, and accuracy of BPAS-MRI combined with TOF-MRA were 95.6%, 95.0%, 93.5%, 96.6%, and 95.2%, respectively, with a kappa value of 0.903. For vertebral artery dysplasia, these metrics reached 100%, 96.6%, 95.8%, 100%, and 98.1%, respectively, with a kappa value of 0.961. In cases of vertebrobasilar artery dissection or dissection aneurysm, the values were 81.8%, 96.8%, 97.8%, 75.0%, and 95.2%, respectively, with a kappa value of 0.756. The proposed BPAS-MRI criteria for the primary causes of vertebrobasilar abnormalities are summarized in Table 9, along with comparisons to conventional MRI.

The main features of the three arteriopathies are summarized in the following list:

(1) Atherosclerosis: TOF-MRA may reveal multiple cerebrovascular stenoses in addition to the stenosis of the vertebrobasilar artery, particularly at vascular bifurcations. There may be evidence of large artery atherosclerosis at other locations, along with findings on conventional high-resolution MRI that are consistent with alterations in the structural features of the vascular wall and a lumen that is characteristic of atherosclerosis [121].

(2) Dysplasia: Both conventional high-resolution MRI and TOF-MRA demonstrate a smoothly narrowed intracranial segment of the vertebral artery, with a diameter reduced to less than half of that of the contralateral side or measuring less than 2 mm. Conventional high-resolution MRI reveals a normal vascular wall structure without any thickening [122].

(3) Dissection: TOF-MRA may indicate the eccentric stenosis of the intracranial segment of the vertebrobasilar artery, presenting with local and segmental stenosis, as well as double-chamber signs. Findings on conventional high-resolution MRI are consistent with changes in the vascular lumen and wall associated with artery dissection or dissecting aneurysms, as noted by Wang et al. [123].

Multisection, Motion-Sensitized, Driven Equilibrium (MSDE) is an advanced MRI technique that shares similarities with diffusion-weighted imaging (DWI). As a preparatory sequence, MSDE begins with a diffusion pulse that uses a very low b-value. The pulse sequence involves three nonselective RF pulses with flip angles of 90°–180°–90° and symmetric gradients surrounding the 180° pulse. MSDE causes the phase dispersion of blood spun through the application of a magnetic field gradient, which effectively suppresses the blood flow signal. This enables the acquisition of 3D T1 or T2-weighted images* [124]. MSDE is particularly useful in various applications, including the detection of brain tumors, carotid arterial atherosclerotic plaques, and small structures like thin cranial nerves [125,126]. Intramural hematomas appear as regions of hyperintensity in this modality because MSDE can cancel any flow signal without an in-flow effect. Because thin-slice images (0.8 mm) can be obtained, MSDE enables us to delineate small intramural hematomas as high-intensity lesions in small arteries [104].

**Figure 3:**
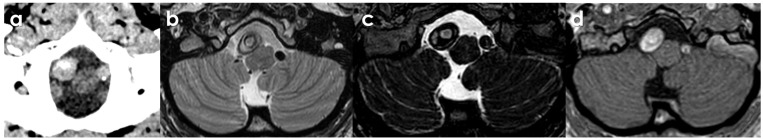
Panel (**a**) shows NCCT, highlighting the right V4 VA-positive remodeling with a fusiform aneurysm characterized by an irregular hyperdense signal into the putative lumen on the axial plane. In panels (**b**–**d**), MRI shows the mural hematoma in the axial view [T2W TSE (**b**), T2W Drive (**c**), T1W Dixon (**d**)] confirming the different ages of the adjacent concentric strata of the hematoma and the sub-acute timing of the dissection, with a more prominent T2W and T1W hyperintensity.

A recent study [127], aiming to identify high-risk clinical and imaging features for intracranial artery dissection, including mostly intracranial VAD (52/75, 70%), used DSA and vessel wall imaging MR on a 3 T system within 30 days after the onset of neurological symptoms. Each dissection was classified into five different types (Type I: classical dissection; Type II: fusiform aneurysm; Type III: long dissected aneurysm; Type IV: huge aneurysm; Type V: saccular aneurysm) in accordance with previous studies and consensuses [68,128]. Most of the lesions were of classical dissection, followed by saccular and dolichoectatic dissecting aneurysms, while fusiform and long dissected aneurysms were uncommon. Most of the lesions were located in the VA with hematomas (88.9%). Double-lumen signs were visible in 48/75 (64.0%) lesions on MRI. However, these were not visible in more than 40% of the classical dissection cases, which might be the reason why 30% of the classical dissection cases were not visualized by DSA. Intimal flaps were visible in most of the lesions (93.3%). Enhancements on CE-T1 images were observed for most intimal flaps and vessel walls, and the enhancement was also observed for the hematoma. Intracranial artery dissection was diagnosed in 17 out of 75 cases (in 16 of which, this was judged to be the culprit), but these were not visible on DSA and diagnosed only with MRI. Among these, 4 lesions showed normal luminal dimension, 10 narrowing, 2 dilations, and 1 mixture of narrowing and dilation. Most of these cases (n = 15) were Type I (classical dissection), one was Type III (long dissected aneurysm), and one was Type IV (dolichoectatic dissecting aneurysm). Hematomas were visible in 14 cases; intimal flaps were found in 15 cases, and double-lumen signs were found in 10 cases on MRI. Compared with DSA, MRA, or CTA, high-resolution, contrast-enhanced MRI can provide significantly greater detail regarding lesions. For example, by analyzing the signal intensity on T1-weighted and T2-weighted images, hematomas can be classified based on their age: hyperacute (<24 h), acute (1–3 days), early subacute (>3 days), late subacute (>7 days), and chronic (>14 days). If the hematoma appears homogeneous on MRI, it may suggest that the dissection occurred at a single point in time, while a heterogeneous appearance could indicate recurrent development (Figure 3).

With the use of contrast agents, MRI enables assessment of local inflammation and the presence of vasa vasorum through vessel wall enhancement [129,130]. This enhancement also helps differentiate the stage of the lesion [131,132]; for instance, the enhancement tends to decrease from the earlier stages to the chronic stage. Persistent vessel wall enhancement may indicate intimal hyperplasia, granulation tissue, or the development of vasa vasorum during the healing process [133]. However, no significant differences were observed in this study when comparing the enhancement between culprit and non-culprit lesions. Given its advantages in demonstrating luminal stenosis, intramural hematoma, and outer wall boundaries, along with its high accuracy in detecting intracranial arterial dissection and its non-invasive nature, MRI should be implemented as a routine imaging protocol prior to invasive DSA. The main features of MR-based techniques in intracranial dissection are summarized in Table 10.

Angiographic studies reveal that fusiform aneurysms and pearl-and-string lesions are the most frequently observed findings in intracranial VAD presenting with SAH, followed by string lesions [88]. Classical radiological indicators of dissection, such as double lumens and intimal flaps, may not always be evident. When a tapering lesion (string lesion) or a partially circumferential aneurysm is present, it can easily be mistaken for an atherosclerotic plaque or a saccular aneurysm. High-resolution 3 Tesla MRI can aid in distinguishing between atherosclerotic plaque and dissection [141], while MR wall imaging can provide complementary information to assist in diagnosis.

The identification of perforators is critical prior to treating intracranial VAD, but they are not easily visualized on CTA. Three-dimensional digital subtraction angiography (3D-DSA) and reconstructed images from standard systems can enhance their identification [142]. Ultra-high cone beam CT has also emerged as a valuable imaging modality, with recent advancements improving the delineation of the number and anatomy of perforators [143].

Intracranial VADs are classified based on their relationship with the PICA, as well as vertebral dominance and collateral flow. Four subtypes of intracranial VAD can be distinguished: (1) involving PICA; (2) proximal to PICA; (3) distal to PICA; (4) absent PICA in the V4 segment.

A cerebral arterial dissection diagnosis should rely on a combination of CT and MRI. In many patients with clinical manifestations suggestive of cerebral arterial dissection, these imaging modalities are likely to reveal intramural hematomas and double lumens. In younger patients, a primary differential diagnosis is often atherosclerotic plaque, and the available imaging techniques have various limitations that should be taken into account (see Table 11). Additionally, in cases of true arterial dissection, imaging findings may evolve over time.

Recently, an attempt to establish a systematic approach to suspected intracranial VAD without conclusive imaging findings was made [144]. The main premise of this approach is that, with advances in imaging technology, non-invasive MRI has become the primary imaging modality for diagnosing cerebral artery dissection [97,145], but not all patients had conclusive imaging findings [100]. Furthermore, the clinical characteristics and prognoses of those with non-definitive VAD remain largely unexplored. Additionally, it is well established that the morphology of the lesion can undergo dramatic changes during the acute phase, making initial treatment decisions critical to prevent poor outcomes [146]. The recent literature on the diagnostic criteria for VAD has proposed three categories: definitive, probable, and possible. However, there is a scarcity of reports discussing the correlation between imaging findings and prognoses. Pragmatically, the diagnosis of VAD is considered definitive when intramural hematoma (IMH), an intimal flap, and pearl-and-string signs are found in the vertebral artery [101,147] but also if arterial stenosis (the string sign) and/or an irregular vessel wall (the pearl sign) are found on imaging in selected cases when the clinical history and examinations are suggestive of VAD.

**Figure 4:**
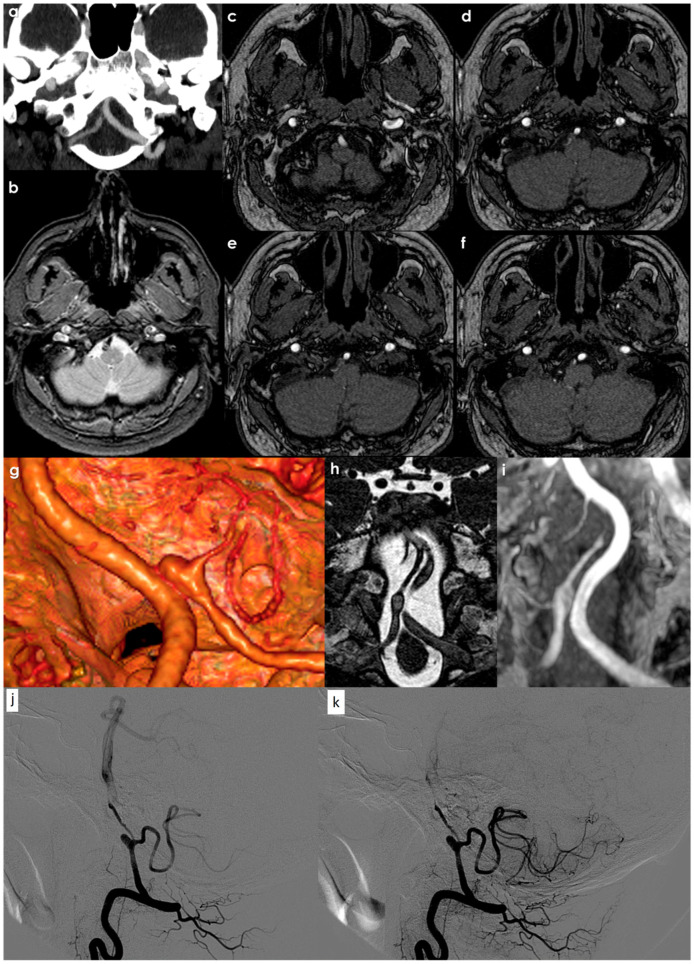
The right V4 VAD involving the takeoff of the PICA in the acute phase: panel (**a**) (CTA reconstructed in a coronal oblique plane with MIP/MPR protocol); panel (**b**) (T2* MRI with the hypointense hematoma causing focal VA occlusion and positive remodeling); panels (**c**–**f**) (MRA source images showing the absence of flow in the dissected segment including the PICA origin). In the 10-day CTA follow-up (panel (**g**)), the partial recanalization of the dissection segment with a PICA aneurysm is shown using a 3D reconstruction in a volume rendering. The corresponding MRI (panel (**h**), balance) and MRA (panel (**i**), coronal plane in MIP/MPR protocol) confirmed this finding, well evident in DSA from right VA injection in lateral view in early (panel (**j**)) and late arterial phase (panel (**k**)). A 20-day follow-up DSA illustrated, in lateral view, the aneurysm at the origin of the left PICA and the severe, irregular stenosis, of the post-PICA in the left V4 VA.

Following an intracranial arterial dissection, some patients may experience a remodeling of their vascular structures, leading to the formation of dolichoectatic or fusiform aneurysms that remain intact without rupturing. The progression of posterior circulation dissecting aneurysms, especially those that arise without accompanying stroke or hemorrhage, was thoroughly examined by Kobayashi et al. [148]. In their investigation involving 113 patients—predominantly asymptomatic or presenting with pain—over an average follow-up of 2.9 years, the researchers observed that 97% of the subjects remained clinically stable, and 80% of the aneurysms exhibited no morphological changes. Only 4% of the cases showed aneurysm enlargement, which correlated strongly with clinical worsening. Hence, it is reasonable to conclude that patients with posterior circulation dissecting aneurysms who do not present with stroke or hemorrhage can typically be treated conservatively, reserving interventional procedures for situations involving mass effects or lesion growth. A rapid process of vessel remodeling and repair is characteristic of both intracranial and extracranial dissection. In Figure 4, an example of the evolution of a VAD is illustrated.

In the context of SAH, the timeline for vessel repair remains unclear, as most patients undergo some form of intervention. However, in cases presenting with ischemia, histological analyses indicate that arteries begin their repair mechanisms within one-day post-dissection. Notably, significant geometric alterations in the luminal appearance of intracranial arterial dissection are observed in over 80% of instances, with substantial changes occurring within the initial two months. Complete arterial normalization is noted in up to 20% of cases and can be detected as early as two weeks following the incident. From a histopathological perspective, necrotic alterations and neutrophil infiltration are evident within 24 h, while newly formed smooth muscle cells and macrophages become visible in the arterial wall within a week [149]. Imaging studies conducted six months after dissection demonstrate recanalization in 20–58% of patients, whereas 30–77% show no variation in the vessel caliber [97,150]. Given that dissections often stem from structural vulnerabilities in the vessel wall, recurrent dissections—especially at the original site—are relatively frequent. A study with an average follow-up of 3.4 years reported a recurrent dissection rate of 9% [151]. This figure surpasses the recurrence rates associated with extracranial dissection, which are cited as 2% within the first month post-diagnosis, followed by 1% annually thereafter [152]. Notably, two-thirds of recurrent dissections manifest within one month of the initial event [151].

## 6. Treatment

Currently, there are no evidence-based protocols for managing intracranial arterial dissection due to its infrequency. The medical approach typically involves antithrombotic or antiplatelet therapy aimed at preventing thromboembolic strokes. Various randomized controlled trials and retrospective analyses have indicated no significant difference in stroke prevention between antiplatelet agents and anticoagulants for patients with cervical artery dissection, and this information has occasionally been applied to intracranial cases [153,154,155,156,157]. However, equivalent studies specifically focusing on IAD are lacking. Intravenous thrombolysis has been regarded as a safe option for patients with cervical arterial dissection experiencing ischemic strokes, leading to the assumption that it may also be safe for those with intracranial dissection, although existing evidence is largely based on individual case reports [156,157]. For patients with intracranial artery dissection leading to large vessel occlusions, there are no prohibitive factors against endovascular recanalization [158]. Nevertheless, these lesions can sometimes mimic active atheromatous plaques, resulting in the short-term reocclusion of the parent artery. Some experts suggest a low threshold for employing stenting in these cases. There will be a segment of patients who experience recurrent strokes despite receiving medical treatment. For these individuals, it may be prudent to explore options such as stent reconstruction using self-expanding stents or surgical bypass procedures. Such strategies have shown a reasonable degree of success in previous case reports and small series. The main issues with the medical treatment of VA dissection are summarized in Table 12.

## 7. Conclusions

Intracranial VAD remains a rare cause of ischemic stroke in young adults. The pathophysiology of spontaneous VAD presenting with ischemic stroke versus a hemorrhage remains unclear, with ongoing debates regarding whether intimal or medial lesions initiate the process. When blood enters the false lumen due to a vessel wall tear, it can lead to various complications, such as subarachnoid hemorrhage, pseudoaneurysm formation, and ischemic symptoms, depending on how the dissection progresses. Histological studies suggest that medial degenerative changes may precede dissection, with a higher incidence of complications in patients presenting with hemorrhage. Clinical symptoms vary widely, with common presentations including headache, neck pain, and ischemic strokes, while prodromal symptoms like dizziness may also occur, complicating the diagnosis and leading to significant neurologic deficits in the affected patients. It can exhibit variable imaging findings, complicating a timely diagnosis and treatment. Currently, there are no standardized diagnostic criteria for VAD; instead, the diagnosis relies heavily on imaging, which can be challenging due to the anatomical variations in the VAs that may obscure the pathological findings. The presence of an intramural hematoma identified through three-dimensional, black-blood, T1-weighted imaging has emerged as the most reliable indicator for diagnosing VAD, but DSA remains fundamental in selected cases. The treatment of patients presenting with acute stroke has not yet been well defined, and a clinical trial on antithrombotic therapy might be useful.

## Figures and Tables

**Table 1:** Course of an intracranial dissection.

Subtype	Features
Transmural Dissection	If the hematoma disrupts the entire vessel wall, a transmural dissection is present. The clinical symptoms will depend on the surrounding structures: ○ Intradural transmural dissection: leads to subarachnoid hemorrhage. ○ Dissection in a venous plexus area (e.g., cavernous sinus or vertebral venous plexus at the atlantal loop): can develop into an arteriovenous fistula. ○ Dissection in soft tissues: leads to the formation of a false aneurysm (extramural hematoma), which may result in a mass effect, stenosis, or the occlusion of the parent vessel.
Subintimal Dissection	If the dissection remains subintimal, a subadventitial hematoma forms within the vessel wall. The clinical consequences depend on the subsequent behavior of this hematoma: ○ Reopening to the true vessel lumen: The clotted hematoma in the false lumen may be washed out, leading to distal embolization. This is the most common pathomechanism in extradural internal carotid artery (ICA) dissection in adults. ○ Growth within the vessel wall: The hematoma can cause the progressive stenosis of the true lumen, potentially leading to ▪ Hemodynamic infarctions due to critical narrowing. ▪ Embolic events from the turbulent flow. ▪ In the intradural portion, growing hematomas can occlude the perforating branches of the dissected parent vessel, resulting in local ischemia. ○ Chronic organization of the hematoma: If the hematoma becomes chronic, it may organize within the vessel wall. The vasa vasorum can sprout into the organizing hematoma, leading to a growing intramural hematoma. Repetitive dissection may lead to the formation of a “giant partially thrombosed aneurysm”.
Clinical Symptoms	
Consequently, the symptoms of arterial dissection can be attributed to • Mass effects. • Ischemia. • Subarachnoid hemorrhages. • In rare cases, a combination of different symptoms may present simultaneously.	

**Table 2:** A summary of the key points regarding VAD and its complications.

Issues	Features
Etiologies	Multiple factors implicated; associated with inconsistent terminology.
Initial Mechanism	Begins as a tear in the intimal lining of the vessel, creating a “false lumen” for alternative blood flow [32,74,75,76].
True Dissection	Result of subintimal extravasation of blood or blood between the intima and media; most common form of extracranial dissection [51].
Intimal Damage	Exposes pro-thrombotic subendothelial vessel wall, which may lead to thrombus formation and potential embolization [76,77].
Dissection Mechanism	High-pressure arterial blood enters the false lumen, dissecting through the tunica media or between layers.
Hematoma Formation	An expanding hematoma accumulates within the vessel wall due to pro-thrombotic subendothelial exposure.
Possible Complications	- Thrombotic phenomena. - Parent vessel occlusion (stenosis or hematoma expansion). - SAH due to adventitial extension.
Pseudoaneurysm Formation	Occurs if dissection involves all three vessel layers, leading to the encapsulation of the extravascular hematoma.
Characteristics of Pseudoaneurysms	- Lack of external elastic lamina. - Fewer elastic fibers in tunica media. - May rupture into subarachnoid space.
Incidence of Pseudoaneurysms	- 28% of posterior circulation aneurysms. - 3.3% of all intracranial aneurysms. - Causative in ~10% of non-traumatic SAH cases.
Rupture Propensity	High; up to 73% of pseudoaneurysms present with SAH; only 27% show bulbar signs or cerebellar ischemia.

**Table 3:** The diagnostic criteria of intracranial dissection, in accordance with the Japanese proposal [101].

Major Criteria	“Double lumen” or “intimal flap” demonstrated on DSA, MRI, MRA, or CTA. “Pearl and string sign” or “string sign” demonstrated on DSA. Pathological confirmation of arterial dissection.
Minor Criteria	“Pearl sign” or “tapered occlusion” demonstrated on DSA. “Pearl and string sign”, “string sign”, or “tapered occlusion” demonstrated on MRA. “Hyperintense intramural signal” (indicative of intramural hematoma) demonstrated on T1-weighted MRI.
Additional Criteria	Change in arterial shape demonstrated on DSA, MRI, MRA, CTA, or duplex ultrasonography. Absence of other causes of arterial abnormalities.
Definite Dissection	The presence of one or more major criteria, or the presence of one or more minor criteria, along with both of the additional criteria.
Probable Dissection	The presence of one or more minor criteria.

**Table 4:** Proposed terminology for imaging diagnostic criteria of intracranial artery dissection.

Diagnostic Criteria
At least one of the three following features must be present:
1. Fusiform or irregular aneurysmal dilation at a non-branching site of an intracranial artery, with at least one of the following: - Intramural hematoma (hyperintense rim on T1-weighted MRI), intimal flap, or double lumen. - Rapid change in morphology on repeated imaging (increase/reduction in size, new stenosis). - Association with focal stenosis (pearl-and-string sign).
2. Long filiform or irregular stenosis of an intracranial artery, with at least one of the following: - Intramural hematoma (hyperintense rim on T1-weighted MRI), intimal flap, or double lumen. - Rapid change in morphology on repeated imaging (increase/reduction in size, new aneurysmal dilation). - Association with fusiform or irregular aneurysmal dilation (pearl-and-string sign).
3. The occlusion of an intracranial artery that recanalizes into either a fusiform or irregular aneurysmal dilation at a non-branching site, or a long filiform or irregular stenosis.

**Table 5:** Proposed grading of imaging diagnostic criteria for intracranial artery dissection.

Grading	Criteria
Definite Intracranial Artery Dissection	- Stenosis or occlusion developing into fusiform/irregular aneurysmal dilation at a non-branching site. - Intramural hematoma, intimal flap, or double lumen. - Pathological confirmation of dissection.
Probable Intracranial Artery Dissection	- Fusiform/irregular aneurysmal dilation and focal, long filiform, or irregular stenosis (pearl-and-string sign) without SAH, or persisting > 1 month after SAH. - Fusiform/irregular aneurysmal dilation at a non-branching site with rapid morphological changes (increase/reduction in size, new stenosis).
Possible Intracranial Artery Dissection	- Fusiform/irregular aneurysmal dilation at a non-branching site without morphological changes on imaging within 6–12 months. - Long filiform or irregular stenosis with reduction in size or disappearance over time.

**Table 6:** Summary of the classical findings of VAD, as demonstrated by DSA.

Finding	Description	Incidence
Pearl-and-String Sign	Represents a dilatation adjacent to the narrowed dissected segment.	Observed in up to 91% of VAD cases [108].
Double Lumen or Rosette	Indicates the presence of two lumens within the dissected vessel.	Reliable angiographic finding for VAD diagnosis [13,38,45].
Fusiform Dilatation	Simple fusiform dilation of the dissected segment.	Common finding in VAD cases.
Delayed Contrast Clearance	The delayed clearance of the contrast from the false lumen of the dissected vessel.	Indicative of dissection.
Abrupt or Tapered “Cut Off”	Observed in cases of vessel occlusion secondary to dissection, where the flow cannot be visualized beyond a certain point.	Characteristic of severe dissection [45,73].

**Table 7:** A summary of the key points related to the evolution and benefits of multidetector computed tomography (MDCT) and CT angiography (CTA) in detecting VAD.

Issue	Features
Technical Evolution	MDCT has facilitated the development of CTA.
Imaging Efficiency	Efficient scanners can image large body segments within seconds of intravascular contrast injection.
Time for Evaluation	CTA evaluation of cranio-cervical circulation can be obtained in less than thirty seconds.
Image Quality	Reconstructed images are of angiographic quality.
Data Acquisition	Unlimited projections can be obtained from one data acquisition, allowing offline interrogation of vasculature.
Emergent Screening	Particularly beneficial for suspected VAD, aiding in the diagnosis of hemorrhage and ischemia.
Time to Treatment	Significant reductions in time to treatment, morbidity, and cost due to combined non-contrast CT and CTA findings.
Findings on CTA	Reliable demonstration of vessel caliber changes, false lumens, fusiform dilatations, and pseudoaneurysms [112,113].
Reliable Diagnostic Criteria	Narrowed, eccentric lumen associated with increased overall diameter of the dissected artery [84].
Key Findings [73]	(1) Focal dilatation. (2) Intimal flap. (3) Increase in outer diameter. (4) Narrowing or occlusion of vessel lumen.
Comparison of Sensitivity	CTA excels in identifying intimal flaps, pseudoaneurysms, and high-grade stenoses compared to MRA [110].
Wall Thickness Sensitivity	An alteration in wall thickness on CTA is a more sensitive criterion than a change in the luminal diameter in VAD [111].
Meta-analysis Findings	Reported sensitivity and specificity for CTA and MRA are fairly similar in detecting carotid and vertebral artery injuries [114].

**Table 8:** MRI and MRA issues in VAD.

MRI for Intramural Hematoma Detection	- Intramural hematomas typically appear as hyperintense regions on T1-weighted imaging (T1WI). - For optimal detection, MRI should be performed using the thinnest possible slices (<4 mm axial images). - High-field MRI systems (≥3-Tesla units) offer particularly high-resolution images, improving diagnostic accuracy.
MRA for Arterial Dissection	- MRA is a non-contrast alternative to conventional catheter angiography, enabling detailed vessel imaging [115]. - High-field 3.0-Tesla MRI systems are preferable due to their superior resolution. - MRA can reveal key angiographic features such as Pearl-and-string sign. String sign (a long-segment stenosis with a sawtooth appearance). Fusiform aneurysmal dilations. Double lumens and intimal flaps (visible on source images with careful evaluation) [105]. - However, caution is necessary as flow artifacts in time-of-flight MRA may mimic true double lumens or intimal flaps.
3D-T1WI for Intramural Hematoma and Intimal Flap	- 3D-T1-weighted imaging (3D-T1WI) is highly sensitive for detecting intramural hematomas and intimal flaps [105,112]. - Signal intensity changes reflect the paramagnetic effects of hemoglobin breakdown: Early (<7 days) and chronic (>2 months) stages: isointense to surrounding tissue. Intermediate stage (7 days–2 months): hyperintense on T1WI, making it highly detectable. - Thin-slice 3D-T1WI overcomes the limitations of conventional thicker-slice T1WI (≥6 mm). - Contrast-enhanced 3D-T1WI (3D-CE-T1WI) further enhances detection of double lumens and intimal flaps. - However, distinguishing small intramural hematomas from lipid-rich atherosclerotic plaques can be challenging. Follow-up MRI is crucial for monitoring hematoma evolution. Particular vigilance is required in young patients with brainstem infarction.
Susceptibility-Weighted Imaging (SWI) in Dissection	- SWI is a high-resolution technique that detects Intracranial hemorrhage. Oxygenation levels in venous blood and thrombi. Microhemorrhages that may be missed on traditional T2*-weighted imaging (T2*WI) [116]. - In arterial dissection, a crescent-shaped dark rim on SWI may indicate intramural hematoma, further aiding diagnosis.

**Table 9:** Imaging features on BPAS-MRI and conventional high-resolution MRI [117].

	Atherosclerosis	Vertebrobasilar Dysplasia	Dissection
TOF MRA	Irregularly stenosis or unclear or invisible.	Smoothly narrowed, diameter < 2 mm, or Invisible.	Eccentric stenosis with local vascular dilation or segmental stenosis with double-lumen signs.
HR-MRI	The lumen was narrow and the wall had annular or eccentric atherosclerotic plaques.	Without any thickening of the vascular wall, the lumen diameter was <1/2 of the contralateral side, or <2 mm, or absent.	Vascular dilation with the double-lumen sign, intimal flap, or intramural hematoma.
BPAS-MRI	Diffuse dilatation with or without a rough wall, or almost normal.	Smoothly narrowed or invisible.	Segmental dilatation or aneurysmal dilatation.

**Table 10:** A summary of the key points related to the use of MRI and MRA in detecting VAD.

Issue	Features
Detection Methods	MRI and MRA offer sensitive, non-invasive means to detect cervical and intracranial dissection [134].
Advantages of MRA	- Reproducibility of technique. - Uniformity between vendors. - Minimal interoperator variation.
Accuracy of Imaging Techniques	Higher accuracy for predicting intracranial VAD with T2-weighted MRI and basi-parallel anatomical scanning (BPAS-MRI) compared to T2-weighted MRI and conventional angiography [135].
Diagnosis of VAD	Depends on the demonstration of intramural hematoma and alteration in the caliber of the patent lumen.
Shapes of Intramural Hematoma	- Curvilinear. - Crescentic (circumferential). - Bamboo-cut. - Band-line. - Spotty [136]
Signal Intensity Changes (T1-weighted)	Changes from isointense/slightly hyperintense in acute settings to hyperintense in subacute and back to isointense in chronic settings.
Fat Suppression	Aids in distinguishing periarterial atheroma from intramural hematoma [137].
Complementary MRI Findings	- Identifiable intimal flap on proton density or T2-weighted images. - Increased vessel diameter compared to normal side. - Double lumen. - Wall and septum enhancement on contrast-enhanced images (3D SPGR) [45].
MRA Findings	Vascular dissection may appear as a tapered or narrowed vessel lumen; medium-to-large pseudoaneurysms or sacculations can be reliably identified with non-contrast MRA [138].
Diagnosis of Exclusion	VAD may sometimes be diagnosed by exclusion when thrombosis occurs.
Sensitivity and Specificity	- MRA: 20% sensitivity, 100% specificity. - MRI: 60% sensitivity, 98% specificity [139,140].
Dynamic Contrast Enhanced MRA (DCEMRA)	Improves the visualization of complex cervical vasculature anatomy; gadolinium-DTPA provides the rapid imaging of the cerebrovascular circulation.
Disadvantages of MRI/MRA	- Lower spatial resolution compared to CTA or DSA. - Larger slice partitions may obscure subtle or short segment dissection. - Complex anatomy of V3 and V4 segments may cause artifactual loss of flow. - Fat-suppression techniques may fail at cervico-thoracic and cranio-cervical junctions, lowering sensitivity.

**Table 11:** Major limitations of imaging modalities.

Modality	Limitation
MRA	The potential misinterpretation of a double lumen as a flow artifact.
T1WI	The risk of mistaking an intramural hematoma for an in-flow effect.
3D-GdT1WI	The possibility of misidentifying a double lumen as a flow artifact.
MSDE	The data may be insufficient to differentiate intramural hematoma from atherosclerotic plaque.
CTA	The potential for misinterpreting a double lumen as a flow artifact.
Angiography	The data may be inadequate for depicting an intramural hematoma.

**Table 12:** A summary of the key points regarding the medical management of VAD.

Issue	Features
Management Approach	A large number of patients with ischemic symptoms from VAD may be managed medically.
Primary Etiology of Ischemia	Thromboembolism is the primary cause of ischemia following VAD, rather than hypo-perfusion [159].
Non-Invasive Imaging	CT and MRI exhibit infarct patterns consistent with thromboembolism [76].
Angiographic Findings	Demonstrates the branch occlusion of intracranial vasculature distal to the dissected vessel, indicative of thromboembolic phenomena.
Anticoagulant Treatment	Some authors recommend avoiding anticoagulants in all intracranial dissections or a performing lumbar puncture to rule out SAH before treatment [76,160].
Aspirin Treatment	Majority of intracranial VAD patients treated with Aspirin (300 mg/day for 3–6 months); stopped upon evidence of recanalization.
Outcomes by Treatment	- Aspirin: 82% favorable outcomes. - Aspirin/Warfarin: 77%. - Heparin/Warfarin: 8%.
Heparin to Warfarin Bridge	Reported favorable outcomes in non-aneurysmal intracranial VAD; minimal cases of hemorrhage [151,161].
Monitoring Without Intervention	Limited studies; in Mizutani’s study, 155 of 190 patients with intracranial dissection involved vertebral artery [151].
Unruptured Cases	54 unruptured cases with infarction treated with antiplatelet or anticoagulant; others followed with blood pressure control [162].
Complications in Follow-Up	One patient died from the rupture of a dilated IAD and another from brainstem infarction; 18 patients had recurrent dissection [151].
Inconclusive Data	Existing data are inconclusive due to lack of randomized trials, variable treatment algorithms, and differing rates of treatment-associated hemorrhages.
Recommendations	- Treat patients without pseudoaneurysms or significant stenosis with antiplatelet therapy. - Endovascular or surgical intervention for continued thromboembolic symptoms despite antiplatelet therapy.
Need for Further Research	A randomized controlled trial is needed to clarify the best medical therapy, requiring a large sample size.

## Data Availability

No new data were created in this paper.
